# Prior Heterologous Flavivirus Exposure Results in Reduced Pathogenesis in a Mouse Model of Zika Virus Infection

**DOI:** 10.1128/JVI.00573-21

**Published:** 2021-07-26

**Authors:** Mariah Hassert, Tara L. Steffen, Stephen Scroggins, Abigail K. Coleman, Enbal Shacham, James D. Brien, Amelia K. Pinto

**Affiliations:** a Department of Molecular Microbiology and Immunology, School of Medicine, Saint Louis University, Saint Louis, Missouri, USA; b College for Public Health and Social Justice, Saint Louis University, Saint Louis, Missouri, USA; c Sinquefield Center for Applied Economic Research, Saint Louis, Missouri, USA; University of North Carolina at Chapel Hill

**Keywords:** flavivirus, Zika virus, dengue virus, yellow fever, cross-protection, vaccination, heterologous virus

## Abstract

The 2015/2016 Zika virus epidemic in South and Central America left the scientific community urgently trying to understand the factors that contribute to Zika virus pathogenesis. Because multiple other flaviviruses are endemic in areas where Zika virus emerged, it is hypothesized that a key to understanding Zika virus disease severity is to study Zika virus infection in the context of prior flavivirus exposure. Human and animal studies have highlighted the idea that having been previously exposed to a different flavivirus may modulate the immune response to Zika virus. However, it is still unclear how prior flavivirus exposure impacts Zika viral burden and disease. In this murine study, we longitudinally examine multiple factors involved in Zika disease, linking viral burden with increased neurological disease severity, weight loss, and inflammation. We show that prior heterologous flavivirus exposure with dengue virus type 2 or 3 or the vaccine strain of yellow fever provides protection from mortality in a lethal Zika virus challenge. However, reduction in viral burden and Zika disease varies depending on the infecting primary flavivirus; with primary Zika virus infection being most protective from Zika virus challenge, followed by dengue virus 2, with yellow fever and dengue virus 3 protecting against mortality but showing more severe disease. This study demonstrates the variation in protective effects of prior flavivirus exposure on Zika virus pathogenesis and identifies distinct relationships between primary flavivirus infection and the potential for Zika virus disease.

**IMPORTANCE** The emergence and reemergence of various vector-borne diseases in recent years highlights the need to understand the mechanisms of protection for each pathogen. In this study, we investigated the impact of prior exposure to Zika virus, dengue virus serotypes 2 or 3, or the vaccine strain of yellow fever on pathogenesis and disease outcomes in a mouse model of Zika virus infection. We found that prior exposure to a heterologous flavivirus was protective from mortality, and to varying degrees, prior flavivirus exposure was protective against neurological disease, weight loss, and severe viral burden during a lethal Zika challenge. Using a longitudinal and cross-sectional study design, we were able to link multiple disease parameters, including viral burden, with neurological disease severity, weight loss, and inflammatory response in the context of flavivirus infection. This study demonstrates a measurable but varied impact of prior flavivirus exposure in modulating flavivirus pathophysiology. Given the cyclic nature of most flavivirus outbreaks, this work will contribute to the forecasting of disease severity for future outbreaks.

## INTRODUCTION

Zika virus (ZIKV) made a devastating impact when it was introduced in the Americas in 2015 and was declared a public health emergency by the World Health Organization (WHO) (1). During this ZIKV epidemic, it was reported that nearly 800,000 people in the Americas had either suspected or confirmed cases of ZIKV infection (2). While the population in the Americas was naive to ZIKV, multiple flaviviruses including yellow fever virus (YFV) and the four serotypes of dengue virus (DENV1 to 4) are endemic to the area (3, 4). With the introduction of ZIKV into the flavivirus endemic areas of the Americas, the question of how and if prior flavivirus exposure could impact the course of disease with a subsequent flavivirus has become one of the most outstanding questions in flavivirus biology.

The *Flavivirus* genus consists of a number of related arthropod-borne viruses (arboviruses), which represent a substantial burden to global health and economic stability. Flaviviruses are small enveloped positive-stranded RNA viruses in the family *Flaviviridae*. Following entry into susceptible cells, viral replication occurs in the cytosol (5). The flavivirus genome is contained within a single open reading frame, which encodes a single polyprotein. The polyprotein is cleaved into 10 proteins: three structural proteins, the capsid (C), premembrane/membrane (prM/M), and envelope (E), as well as seven nonstructural proteins (6). As the structure and replication of flaviviruses are thought to be highly similar, our understanding of intra and extracellular pathways of flavivirus replication cycles comes from studies of multiple different flaviviruses (reviewed in references 5–8). Similarly, studies have long used existing knowledge of flavivirus structure to build and support the structural studies of emerging flaviviruses, including ZIKV (9–11). The high degree of relatedness between flaviviruses has provided a foundation for understanding emerging flaviviruses but has also confounded epidemiological studies and diagnostics, as the high degree of relatedness makes flaviviruses more difficult to serologically distinguish *in vivo*.

The high degree of genetic and structural similarities between flaviviruses (12) has led to the hypothesis that prior flavivirus exposure could afford some cross-protection against a novel circulating flaviviruses like ZIKV. The dengue literature provides some evidence for cross-protection, with a single DENV serotype showing short-term protection against infection with heterologous serotypes (13). Additionally, multiple studies have recently been published looking at the impact of prior dengue infection on the generation of adaptive immune responses to ZIKV, noting that cross-reactivity exists for both the T cells and antibody responses (reviewed in references 14–17). Based on longstanding literature with DENV (18), there is also substantial concern surrounding potential impact of a prior flavivirus exposure on enhancing disease severity of a subsequent ZIKV infection. There is a well-established link between prior DENV exposure and enhanced disease during infection with a heterologous DENV serotype (18). With the introduction of ZIKV into areas of South and Central America where DENV and YFV are endemic, there is a question regarding whether prior exposure to a heterologous flavivirus could enhance disease severity. However, what is missing from these studies, and field studies of ZIKV infection in the Americas, is the ability to systematically monitor infection and ZIKV disease course in the context of prior flavivirus infection. So, while epidemiological studies have provided excellent insight into potential correlations between prior heterologous flavivirus exposure and ZIKV pathogenesis, murine models of heterologous infection, where more invasive systematic studies can be completed, are important for testing potential correlations provided by field studies.

What we have learned from animal studies and clinical observations of flavivirus infection is that the high degree of similarity in flavivirus replication cycles, genetics, and structures does not necessarily translate into similarities in cell tropism, pathogenesis, disease course, and outcomes following infection. An example of this would be the comparison between YFV and ZIKV. ZIKV infection is primarily asymptomatic in adults and children and has shown significant tropism for the central nervous system (CNS); where YFV is primarily thought of as a hemorrhagic fever virus causing mild to severe disease in 45% of those infected and can have a mortality rate as high as 8% (WHO). So, while we have been able to use the strong structural studies of flaviviruses including DENV and YFV to make rapid advancements in our understanding of ZIKV biology, we have had to rely more heavily on animal models to understand the implications for disease. This is especially true in addressing the question of how a prior flavivirus infection impacts the disease course of a subsequent flavivirus infection, where prior studies have relied heavily on the structural and immunological similarities between flaviviruses to predict disease outcomes.

Small animal models of infection have been critical for defining the correlates of protection and modeling disease for several flaviviruses, including DENV (19), YFV (20, 21), and ZIKV (22–24). In the case of ZIKV, the type 1 interferon receptor knockout mouse model (Ifnar1^−/−^) has been used extensively for these purposes. A few studies in murine systems have demonstrated that prior infection with a mouse-adapted strain of DENV2 (strain D2S20) protects from a lethal ZIKV challenge (25) and to some degree is protective from fetal loss in maternal infection models (26). However, whether ZIKV cross-protection is similar, independent of prior flavivirus infection, is unclear. In addition, while murine models have shown that prior DENV2 infection prevents mortality, the impact of prior flavivirus exposure on pathogenesis and disease course still remains unclear, namely, the consequences for viral neuroinvasion and physical indicators of neurological disease.

To address the gaps in knowledge of heterologous flavivirus exposure, we have completed a comprehensive longitudinal cross-protection study, collecting multiparametric data to evaluate the impact of prior exposure to ZIKV, DENV2, DENV3, and the vaccine strain of YFV (YF-17D) on ZIKV neurological disease. With this study, we demonstrated that a sublethal heterologous flavivirus exposure confers varying degrees of protection from ZIKV mortality, weight loss, neurological disease, and excessive cytokine production in the CNS. Prior exposure to either ZIKV, DENV2, DENV3, or YF-17D significantly reduced viral burden in the spleen, liver, kidney, brain, and spinal cord of mice infected with ZIKV in comparison to a primary ZIKV infection. Importantly, there were significant differences in the degrees of protection afforded by heterologous flaviviruses, with prior exposure to either ZIKV or DENV2 being the most protective from ZIKV challenge. Exposure to YF-17D or DENV3 lessened mortality, disease severity, as well as duration of disease, though some animals still succumbed to infection. These data demonstrate that prior heterologous flavivirus exposure has a significant but varied effect on ZIKV replication and disease burden. The results of this study suggest that, along with cross-reactive immune responses, infection history can influence flavivirus disease course.

## RESULTS

### DENV, ZIKV, and YFV share a significant degree of geographic and genetic overlap.

**FIG 1:**
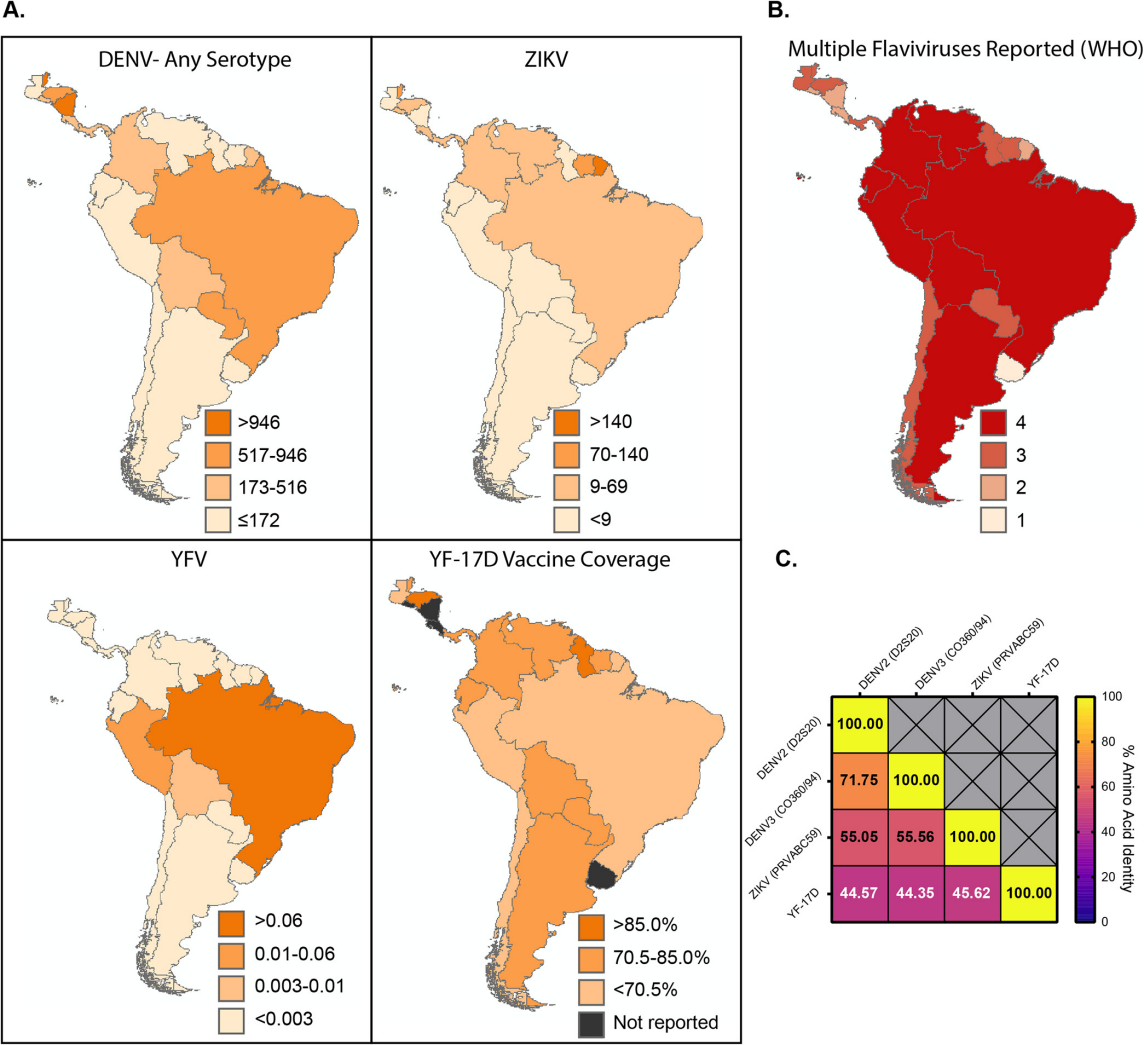
The flaviviruses contained within this study share a substantial degree of geographic and genetic overlap. (A) Average annual incidence rates per 100,000 people in South and Central American countries from 2015 to 2019. DENV, ZIKV, and YFV infections and YF-17D vaccine coverage were reported by the WHO/PAHO. Data is displayed as annual average incidence per 100,000 people in a given country for infections or percent reported vaccine coverage for YF-17D. (B) The number of each of the flaviviruses of interest reported in each country from 2015 to 2019 ranging from 1 to 4. (C) Amino acid identity of the full-length polyprotein of each virus used in the current study.

Many areas in South and Central America are considered endemic for circulation of flaviviruses including YFV and the four serotypes of DENV (WHO/Pan American Health Organization [PAHO]). ZIKV, DENV, YFV, and YF-17D vaccine coverage collected from case incidence data from the WHO/PAHO database for South and Central American countries from 2015 to 2019 (Fig. 1A) demonstrate the relative infection and vaccination rates for the flaviviruses during and following the ZIKV epidemic. Most people living in these regions had likely never been exposed to ZIKV prior to the 2015/2016 ZIKV outbreak (27), yet by 2019, ZIKV was endemic in most South and Central American countries. In 2017, as ZIKV infections continued to be documented, the northern region of Brazil was experiencing a surge of YFV cases, prompting a large vaccination campaign. Additionally, the reemergence and dramatic increase in DENV cases in South America over the last 50 years has resulted in many regions in South America being termed “dengue hyperendemic” (3). These most recent outbreaks are reflected in the WHO/PAHO data and demonstrate that the vast majority of South and Central American countries have reported the circulation of more than one of these flaviviruses over the past 5 years (Fig. 1B). The cyclic nature of the endemic flavivirus infections within this region suggests that even in years where disease incidence is relatively low, the prior flavivirus exposure rate within the population is relatively high (28, 29). Given the recent ZIKV outbreak and the high likelihood that ZIKV, or another flavivirus, will reemerge and cause disease, the question of how prior flavivirus exposure influences infection and disease of a subsequent flavivirus is crucial.

There are a significant number of genetic and antigenic similarities between ZIKV, the four serotypes of DENV, and YFV (12, 30). Comparison of the amino acid identity of the full-length polyproteins between ZIKV (strain PRVABC59), DENV2 (strain D2S20), DENV3 (strain CO360/94), and YFV (strain YF-17D) demonstrates between 44% to 71% identity between the viruses in various combinations (Fig. 1C). Based on epidemiological studies demonstrating the potential for immune mediated cross-protection (29, 31) as well as extensive genetic overlap (Fig. 1C) (32), we hypothesized in this study that exposure to DENV or YFV would confer some protection from ZIKV pathogenesis in a murine model.

### Prior flavivirus exposure impacts ZIKV disease progression and mortality.

**FIG 2:**
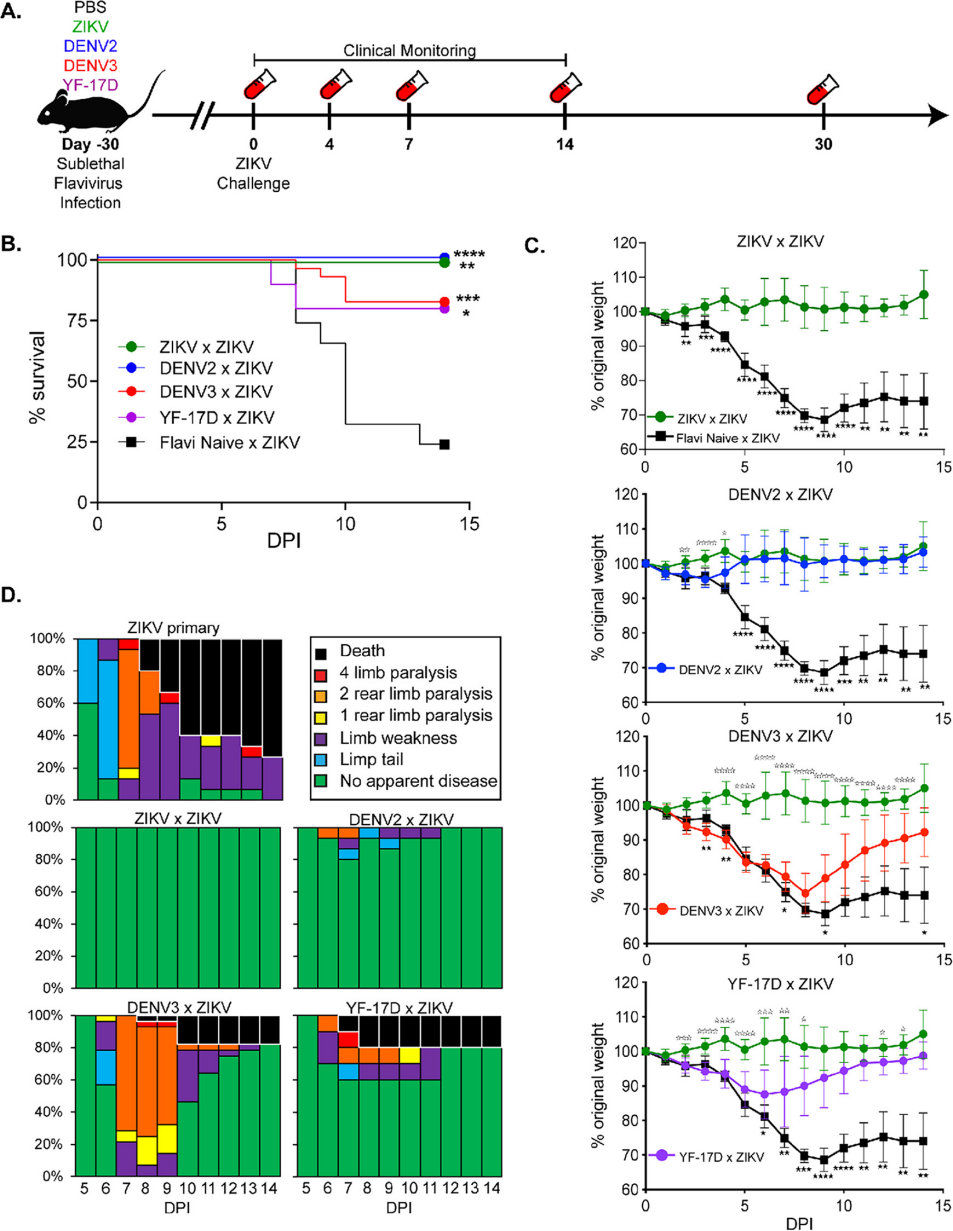
Prior flavivirus exposure leads to reduced disease severity and mortality during ZIKV challenge. (A) Experimental design. Ifnar1^−/−^ mice were sublethally infected with either ZIKV (*n* = 8), DENV2 (*n* = 15), DENV3 (*n* = 30), YF-17D (*n* = 10), or PBS as a flavivirus-naive control (*n* = 11). Thirty days following primary infection, mice were challenged with ZIKV by i.v. administration. For 14 days following ZIKV challenge, mice were monitored for indicators of neurological disease as previously described (22, 23), weight loss, and mortality. At days 4, 7, 14, and 30 post ZIKV challenge, blood was collected to measure viremia by qRT-PCR. Data is a compilation of 4 independent experiments with at least 8 animals per group. (B) Survival of Ifnar1^−/−^ mice with or without prior flavivirus exposure during i.v. ZIKV challenge. Statistically significant differences in survival rates were determined by Mantel-Cox test (*, *P* = 0.03; **, *P* = 0.002; ***, *P* = 0.0002; ****, *P* < 0.0001). (C) Weight loss during ZIKV challenge. As a measure of disease burden, mice were weighed daily for 14 days post ZIKV challenge. Weight change is displayed by normalizing relative to the starting weight of each animal on the day of ZIKV challenge. Statistical significance in weight loss over time was determined by two-way ANOVA with Dunnett’s *post hoc* analysis (*, *P* = 0.03; **, *P* = 0.002; ***, *P* = 0.0002; ****, *P* < 0.0001). Closed stars represent statistical significance from flavi-naive × ZIKV, and open stars demonstrate statistical difference from ZIKV × ZIKV. (D) Neurological indicators of ZIKV disease. Mice were evaluated daily for sequela associated with ZIKV infection and graphed as a percentage of the total number of mice per group.

To examine the cumulative effects of prior flavivirus exposure on protection from ZIKV through longitudinal study, sequential challenge experiments were performed in a mouse model of infection and pathogenesis (24, 33). Four- to five-week-old Ifnar1^−/−^ female and male mice were vaccinated intravenously (i.v.) with 10^5^ focus-forming units (FFU) of either DENV2 (mouse-adapted strain D2S20) or DENV3 (strain CO360/94). Eight-week-old Ifnar1^−/−^ mice were vaccinated subcutaneously (s.c.) with 10^5^ FFU of the vaccine strain of YFV (YF-17D). As positive and negative controls for this experiment, 8-week-old female and male Ifnar1^−/−^ mice were vaccinated with 10^5^ FFU of ZIKV (s.c.) or phosphate-buffered saline (PBS), respectively. Primary viral infection was confirmed by reverse transcriptase PCR (RT-PCR) from bleeds 3 days post primary infection (data not shown). All primary viral infections were given to mice at specific ages and at specific doses and routes of infection that were sufficient to result in infection but not mortality (19, 21–23). Approximately 30 days following primary infection, flavivirus-immune and -naive Ifnar1^−/−^ mice were challenged with 10^5^ FFU of ZIKV i.v. route. We have previously established that this, in a naive animal, is a lethal route of infection, resulting in 80 to 90% of mice succumbing to infection by day 14 (22, 23). During the i.v. ZIKV challenge, the mice were then monitored daily for 14 days for mortality and indicators of disease, such as weight loss and limb weakness or paralysis. At multiple time points following ZIKV infection (days 4, 7, 14, 30), blood was collected in a subset of mice via cheek bleed and analyzed for viral burden via reverse transcription-quantitative PCR (qRT-PCR) (Fig. 2A).

Consistent with previous literature (34), mice with prior ZIKV exposure (ZIKV × ZIKV) were completely protected from overt ZIKV-induced morbidity and mortality (Fig. 2B to D) compared to mice with no prior flavivirus exposure (flavi-naive × ZIKV) that lost a significant amount of weight (Fig. 2C) and all suffered from ZIKV-induced neurological indicators of disease, including flaccid tail, limb weakness, hind limb paralysis, or complete limb weakness or paralysis (Fig. 2D). Approximately 75% of mice challenged with ZIKV in the absence of prior flavivirus exposure eventually succumbed to infection, consistent with our previous reports (22, 23) (Fig. 2B).

Upon infection with ZIKV, mice with prior heterologous flavivirus exposure had significantly reduced mortality relative to flavivirus-naive mice infected with ZIKV. One hundred percent of mice with prior DENV2 exposure (DENV2 × ZIKV), 80% of mice with prior DENV3 exposure (DENV3 × ZIKV), and 80% of mice with YF-17D exposure (YF-17D × ZIKV) survived ZIKV challenge (Fig. 2B). As we had hypothesized, these data show that prior heterologous flavivirus exposure is protective from ZIKV-induced morbidity and mortality in the Ifnar1^−/−^ mouse model. However, what was surprising was the varying degrees of cross-protection observed depending upon the primary infecting virus.

Comparison of the protection from morbidity afforded by prior sublethal ZIKV infection to that of prior heterologous flavivirus exposure revealed unexpected differences in protection between the flaviviruses (Fig. 2C and D). In the case of DENV2 exposure, these mice lost significantly less weight than the flavivirus-naive mice during ZIKV challenge (flavi-naive × ZIKV), and only on days 2 to 4 post ZIKV challenge did prior DENV2 exposure result in significant differences in weight loss between the mice that had received a prior DENV2 (DENV2 × ZIKV) infection and the mice that had previously seen ZIKV (ZIKV × ZIKV) (Fig. 2C). This mild evidence of disease in the DENV2-immune mice is also seen in the clinical scoring, where only 20% of the mice showed any signs of neurological impairment (Fig. 2D). However, in the case of prior DENV3 exposure (DENV3 × ZIKV), partial protection from ZIKV was conferred as indicated by only a mild reduction in weight loss relative to the flavi-naive × ZIKV group during ZIKV challenge (Fig. 2C). Conversely, comparison of the weight loss between ZIKV-immune and DENV3-immune mice showed a significant weight loss in the DENV3-immune mice starting day 4 post ZIKV infection and continuing until day 13, and all mice within this group displayed some signs of neurological involvement (Fig. 2D), suggesting that these mice were not as resistant to ZIKV-induced weight loss and disease. In examining the protection provided by prior exposure to YF-17D, we saw an intermediate phenotype between DENV2 and DENV3. All of the YF-17D mice lost some weight following ZIKV challenge, with the YF-17D-immune mice showing a significant difference in weight loss compared to the flavi-naive × ZIKV group beginning on day 6 and with a peak weight loss occurring between days 6 and 7 before the mice began to recover (Fig. 2C). Most YF-17D-immune mice did not completely recover to their original body weight by day 14 post ZIKV infection. The neurological disease score showed a similar trend, with 40% of the mice showing evidence of neurological disease (Fig. 2D). These results suggest that prior heterologous flavivirus infection can protect against mortality, and individual heterologous flaviviruses have distinct influences on the severity of morbidity associated with ZIKV.

### ZIKV viremia over time but not ZIKV neutralization is differentially influenced by prior heterologous flavivirus exposure.

**FIG 3:**
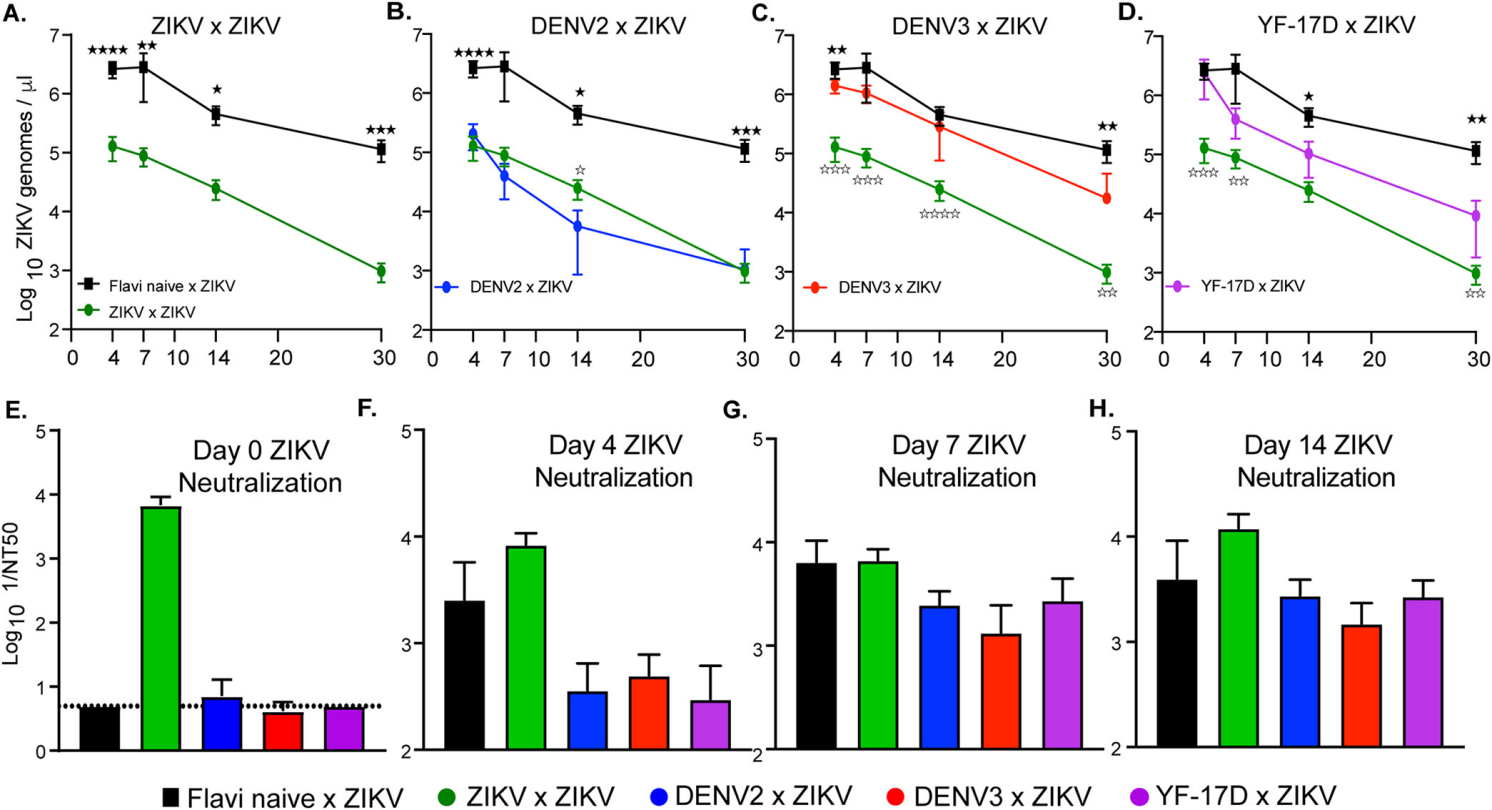
ZIKV viremia over time and ZIKV neutralization potential following heterologous challenge. Following ZIKV challenge, blood was collected via submandibular bleed on days 4, 7, 14, and 30 to evaluate viremia over time by qRT-PCR. (A) ZIKV viremia of homologously primed and boosted (ZIKV × ZIKV) mice compared to that of mice with no prior flavivirus exposure during ZIKV challenge. (B) ZIKV viremia of DENV2-immune mice during ZIKV challenge (DENV2 × ZIKV) compared to that of mice with no prior flavivirus exposure during ZIKV challenge. (C) ZIKV viremia of DENV3-immune mice during ZIKV challenge (DENV3 × ZIKV) compared to that of mice with no prior flavivirus exposure during ZIKV challenge. (D) ZIKV viremia of YF-17D-vaccinated mice during ZIKV challenge (YF-17D × ZIKV) compared to that of mice with no prior flavivirus exposure during ZIKV challenge. (E) At days 0 and 14 post ZIKV challenge, serum was collected and used in a ZIKV focus reduction neutralization test (FRNT) to measure the capacity of serum from each mouse to neutralize ZIKV. Neutralization was determined by enumerating a reduction in infectious particles with increased serum concentration and determining the NT_50_ or serum dilution at which 50% of infectious virus is neutralized. Neutralization potential over time is displayed as 1/NT_50_ in which higher values indicate more effective neutralization. Statistically significant differences in viremia over time were determined by two-way ANOVA with Dunnett’s *post hoc* analysis (*, *P* = 0.03; **, *P* = 0.002; ***, *P* = 0.0002; ****, *P* < 0.0001) with closed stars representing a statistical significance from flavi-naive × ZIKV and open stars demonstrating a statistical difference from ZIKV × ZIKV. Differences in neutralizing antibody were determined by a Mann-Whitney test.

Throughout the course of this longitudinal study (Fig. 2A), whole blood was collected at multiple time points post ZIKV exposure and analyzed by qRT-PCR to assess the impact of heterologous flavivirus exposure on ZIKV viremia over time. Consistent with our previous studies using this model (22, 23), during a primary ZIKV infection (flavi-naive × ZIKV), viral RNA is detectable in the blood by day 4 postinfection and trends in a downward trajectory over time; however, the virus is not cleared even by day 30 in these animals (Fig. 3). Sublethal vaccination with ZIKV prior to ZIKV challenge results in a substantial reduction in viremia (relative to mice with no prior flavivirus exposure) starting as early as 4 days postchallenge and continuing throughout the course of the experiment (Fig. 3A). This is consistent with previous reports demonstrating the protective capacity of ZIKV immunity upon challenge with ZIKV (34–36).

Similar to the influence of prior heterologous flavivirus infection on ZIKV neurological disease and weight loss, viremia was variable and dependent upon the virus that was given upon primary infection. In mice with prior DENV2 exposure, ZIKV viremia was statistically lower throughout the course of infection, with the exception of day 7, relative to mice with no prior flavivirus exposure and was not dramatically different from mice that had received a prior ZIKV infection (Fig. 3B). However, in mice with prior DENV3, only on days 4 and 30 was there a statistically significant reduction in ZIKV viremia relative to a primary infection (Fig. 3C). Comparison of ZIKV viremia from DENV3-immune mice to that of mice that had previously received ZIKV demonstrated significant differences at all time points tested. An intermediate phenotype was observed in the YF-17D-immune mice, where at early time points post ZIKV infection, viremia was significantly higher in the YF-17D-immune mice than in the ZIKV-immune mice, and by day 14 postinfection, viremia was lower in the YF-17D-immune mice than in the ZIKV-naive group. These results demonstrate that prior heterologous flavivirus exposure results in a significant reduction in detectable viral genome copies by day 30 post ZIKV infection.

In the context of a primary ZIKV infection, multiple murine studies have linked protection from disease and reduction in viral burden to a robust neutralizing antibody response (37, 38). Moreover, there have been numerous reports of flavivirus antibody cross-reactivity as measured by enzyme-linked immunosorbent assay (ELISA) in humans, nonhuman primates, and murine models of infection (37–42). One hypothesis would suggest that the observed cross-protection, in the form of reduction in viral burden, may be mediated by cross-neutralizing antibodies generated during the primary heterologous flavivirus exposure. To test this hypothesis, serum was collected from mice during heterologous ZIKV challenge at days 0, 4, 7, and 14 post-ZIKV challenge. The serum was used to evaluate the impact of prior heterologous flavivirus exposure on ZIKV neutralization potential of polyclonal serum samples during a secondary infection via focus reduction neutralization test (FRNT) (39). Using this technique, we determined the concentration of serum at which 50% of ZIKV particles were neutralized (NT_50_) (Fig. 3E to H and Table 1). The inverse of this value was used to indicate serum neutralization potential, with greater values indicating greater neutralization.

**TABLE 1:**
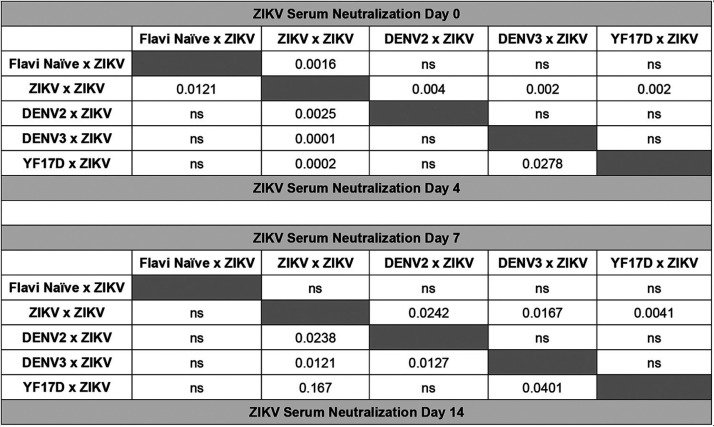
Statistical comparison between ZIKV serum neutralization capacity at days 0, 4, 7, and 14 post ZIKV challenge for each group^*a*^

a On each day, serum was collected and used in ZIKV FRNT. 1/NT_50_ values were compared between each group by Mann-Whitney test. Significant *P* values are indicated for comparison between the groups; ns is used to denote the comparison were the *P* value was above 0.05.

As expected, mice with no prior flavivirus exposure exhibited no neutralizing antibodies against ZIKV prior to challenge, mice with prior ZIKV exposure had high ZIKV-neutralizing antibody titers at the start of lethal ZIKV challenge, and over the course of ZIKV infection the mice that had received a prior ZIKV challenge maintained a significantly higher level of neutralization than the mice that had received a prior heterologous flavivirus infection (Fig. 3E to H and Table 1). Mice with prior exposure to DENV2, DENV3, or YF-17D began with little to no detectable neutralizing antibodies against ZIKV (Fig. 3E and Table 1). Interestingly, on days 4 and 7 postinfection, ZIKV-neutralizing antibody responses were not significantly higher and appeared to be slightly lower in the heterologous immune animals compared to those of naive animals challenged with ZIKV (Fig. 3F and G and Table 1). By day 14 following ZIKV infection, the ZIKV-neutralizing antibody response in these animals did not differ from animals in the flavi-naive × ZIKV group (Fig. 3H and Table 1). These data lead to the conclusion that within this model of heterologous infection, ZIKV cross-protection is not primarily mediated by cross-neutralizing antibodies. These findings, however, do not rule out any potential nonneutralizing antibody functions that could be mediating cross-protection, such as complement activation or antibody dependent cellular cytotoxicity (ADCC).

### Global ZIKV viral burden is reduced with prior flavivirus exposure.

**FIG 4:**
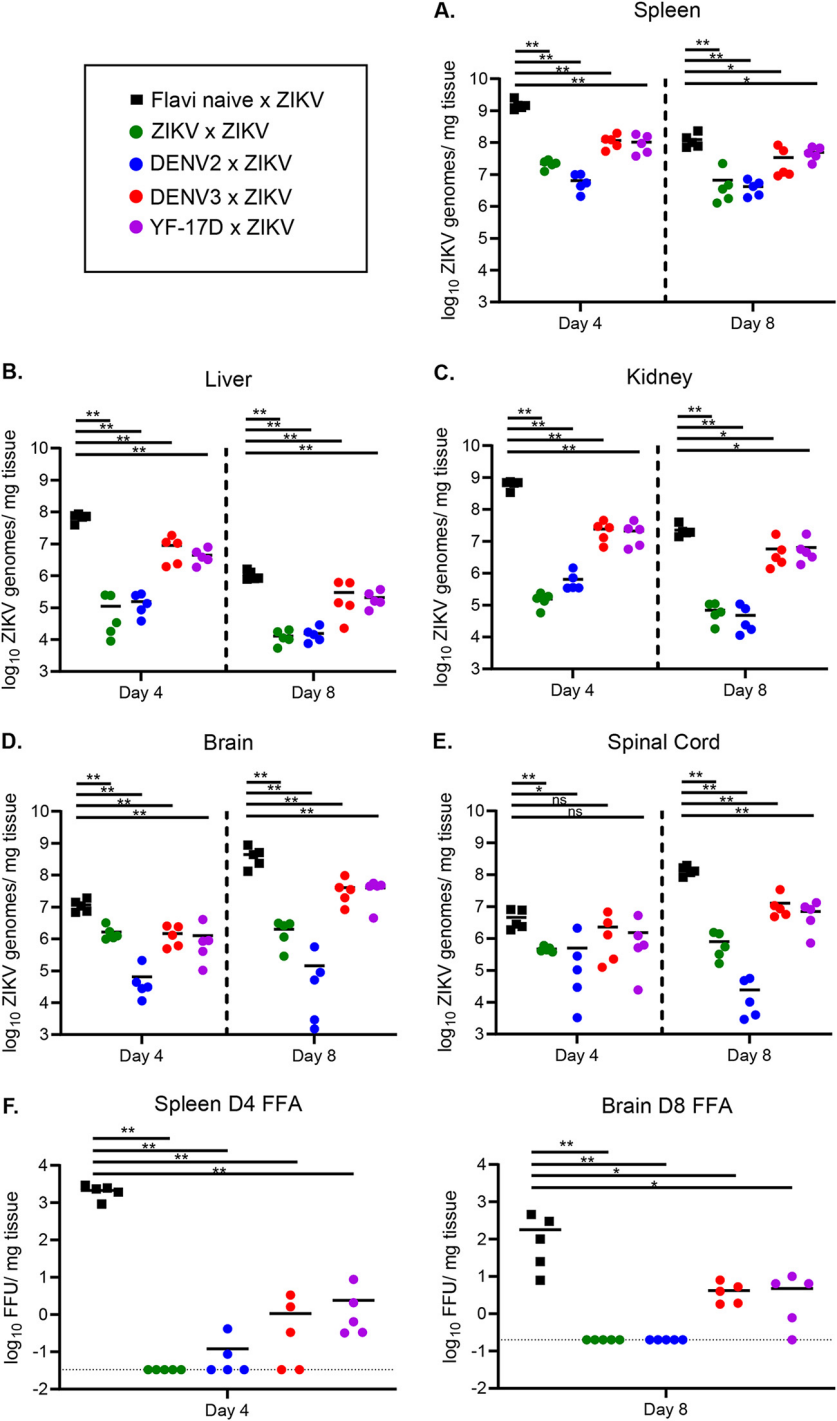
ZIKV viral burden in the peripheral organs and CNS is reduced with prior flavivirus exposure. Ifnar1^−/−^ mice were sublethally infected with either ZIKV (*n* = 10), DENV2 (*n* = 10), DENV3 (*n* = 10), YF-17D (*n* = 10), or PBS as a flavivirus-naive control (*n* = 10). Thirty days following primary infection, mice were challenged with ZIKV by i.v. administration. At days 4 and 8 post ZIKV challenge (*n* = 5 mice per group per day), mice were euthanized, perfused with PBS, and organs were weighed and snap-frozen. RNA was extracted, and qRT-PCR was performed to measure viral burden in the spleen (A), liver (B), kidney (C), brain (D), and spinal cord (E). (F) Infectious virus was quantified in the spleen and brain homogenates of infected mice on days 4 and 8 post ZIKV challenge, respectively, via a standard FFA. Data are displayed as Log_10_ ZIKV genome copies per milligram of tissue. Statistical significance was determined by Mann-Whitney test (*, *P* = 0.03; **, *P* = 0.002; ***, *P* = 0.0002; ****, *P* < 0.0001).

While we observed a reduction in ZIKV viremia over time in mice that had been previously exposed to another flavivirus, it was unclear if heterologous flavivirus exposure could influence viral burden in tissues both within the peripheral organs and central nervous system (CNS). ZIKV invasion of the CNS appears to be a crucial factor in disease pathology within murine models of infection, as well as in human disease (23, 24, 34, 43), although the mechanism that drives this is not fully understood. To determine the impact of prior heterologous flavivirus exposure on the viral burden in various organs during ZIKV infection, we sublethally infected mice with either ZIKV, DENV2, DENV3, YF-17D, or a PBS control. Thirty days postinfection, the mice were challenged i.v. with ZIKV as described in Fig. 2A. At days 4 and 8 post ZIKV challenge, the spleen, liver, kidney, brain, and spinal cord were harvested and homogenized in complete Dulbecco modified Eagle medium (DMEM). A ZIKV focus forming assay (FFA) was completed on organ homogenates, and RNA was extracted from these homogenates to determine ZIKV viral burden by qRT-PCR (Fig. 4).

Similar to previous reports of primary ZIKV infection in the Ifnar1^−/−^ model (23, 24, 33), day 4 is the peak in viral burden in most peripheral organs (Fig. 4A to C) while day 8 is the peak for viral burden in CNS tissues (Fig. 4D and E). By the time of peak neurological pathology in mice with no prior flavivirus exposure (days 8 and 9), the virus has invaded both the brain and spinal cord and replicated to high titers. As expected, mice with prior ZIKV exposure display significantly reduced viral burden in the spleen (Fig. 4A), liver (Fig. 4B), kidney (Fig. 4C), brain (Fig. 4D), and spinal cord (Fig. 4E) relative to flavi-naive × ZIKV mice on both days 4 and 8. However, we find it important to note that this significant reduction does not appear to be completely sterilizing, as viral genomes are still being detected on both days in all tissues. We have previously reported that ZIKV is a persistent infection in the Ifnar1^−/−^ model (23), which could potentially be the reason for this observation.

In the case of mice with prior heterologous flavivirus exposure (DENV2, DENV3, or YF-17D), we saw a significant reduction in ZIKV viral burden in peripheral tissues on both days 4 and 8 relative to that of the flavi-naive × ZIKV group (Fig. 4A to C). The most drastic of these reductions came from mice with prior DENV2 exposure, which trends with the observation of reduced pathogenesis (Fig. 2 and Table 2). On day 4 postinfection, we observed a significant reduction in ZIKV viral load in the brains of all groups of mice with prior flavivirus exposure (Fig. 4D and Table 2). However, in the spinal cord, only heterologously challenged mice with prior DENV2 exposure had statistically significantly reduced viral loads on day 4 (Fig. 4E). By day 8, the peak in disease burden and viral burden in the CNS, all groups of heterologously challenged mice displayed reduced viral load in both the brain and spinal cord (Fig. 4D and E and Table 2). As was seen in Fig. 2, the heterologously challenged mice displayed considerable variation in the control of ZIKV in the periphery and CNS, with the DENV2-immune mice controlling virus as well if not better than the ZIKV-immune mice, and the DENV3- and YF-17D-immune mice controlling virus slightly better than the flavi-naive × ZIKV challenge but significantly worse than the DENV2- or ZIKV-immune mice.

**TABLE 2:**
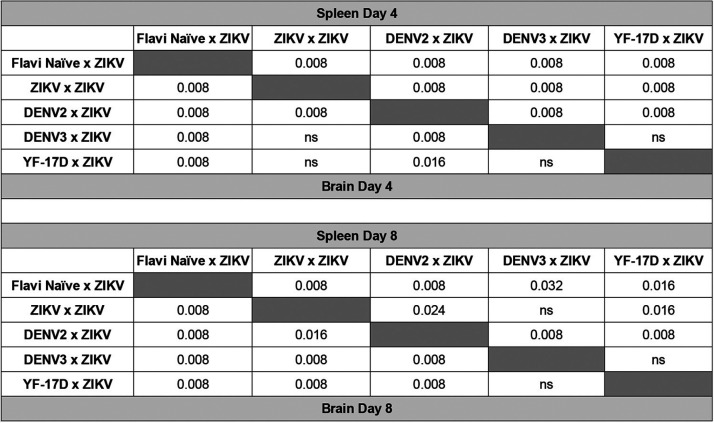
Statistical comparison between ZIKV titers measured by qRT-PCR in the spleens and brains at days 4 and 8 post ZIKV challenge^*a*^

a Viral load was compared between each group by Mann-Whitney test. Significant *P* values are indicated for comparison between the groups; ns is used to denote the comparison were the *P* value was above 0.05.

To confirm that the differences that we observed in viral burden via qRT-PCR translated to analogous differences in infectious virus, we performed focus forming assays (FFA) to measure infectious virus in the spleen and brain homogenates at the peak of viral replication in each tissue (day 4 and day 8, respectively) (Fig. 4F). Consistent with the qRT-PCR data, we found high levels of infectious virus in the flavivirus-naive × ZIKV group in both the spleen and brain on these days. Infectious virus was not detected above the limit of detection in the brain or spleen of mice from the ZIKV × ZIKV group. The level of viral replication was significantly reduced, though still detectable in most animals in the DENV3 × ZIKV and YF-17D × ZIKV groups in the spleen and brain. Infectious virus was not present above the limit of detection in the brains of the animals in the DENV2 × ZIKV group and only above the limit of detection in the spleens of 2 animals in this group. Collectively, this data demonstrates that prior heterologous flavivirus exposure impacts the outcome of ZIKV challenge by a global reduction in viral burden on days 4 and 8 postinfection.

### During heterologous infection, mice display reduced expression of proinflammatory cytokines in the CNS relative to mice experiencing a primary infection.

**FIG 5:**
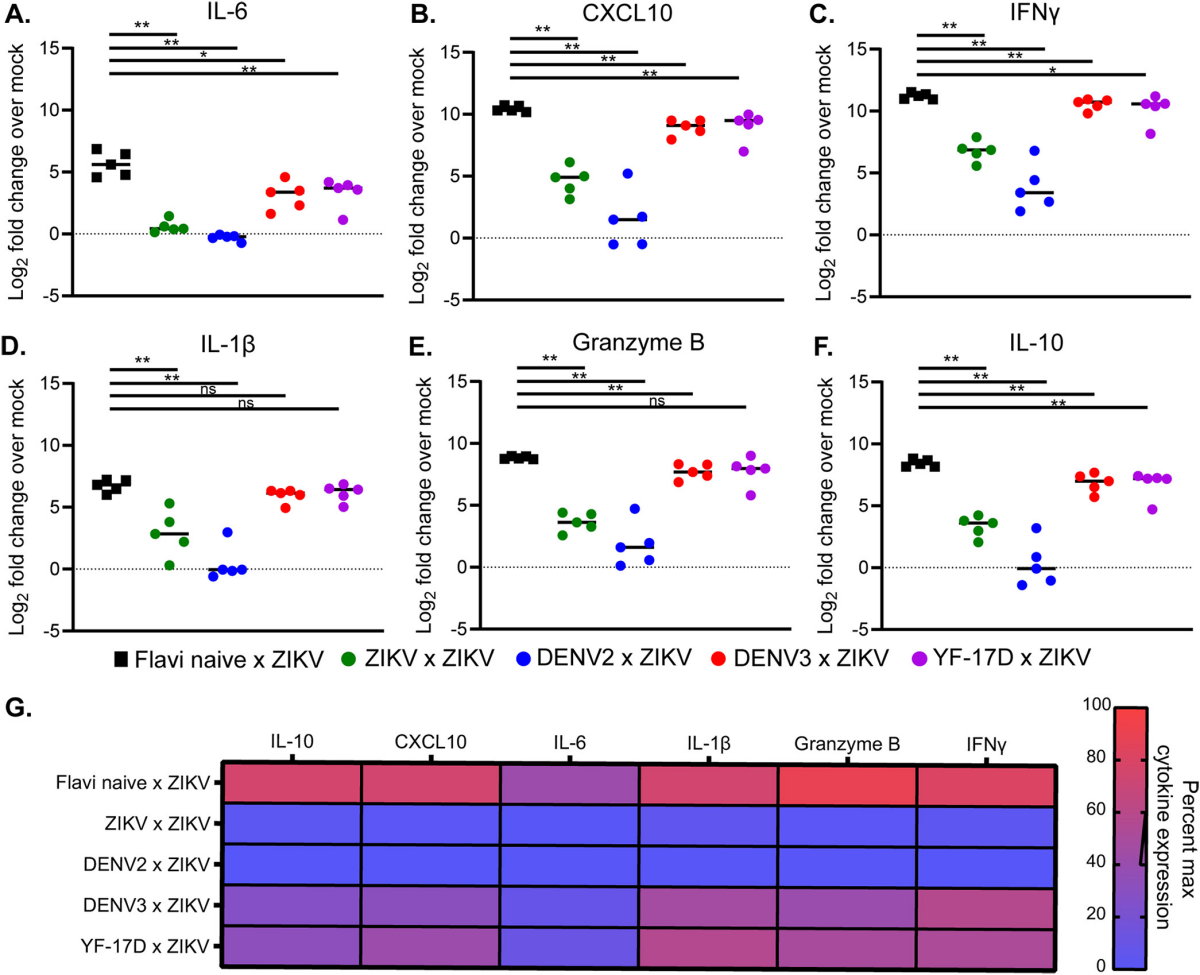
Cytokine expression profiles in the brain during heterologous infection. At day 8 post ZIKV challenge, RNA was isolated from the brains of infected mice or a naive control group (*n* = 5 per group) (the same mice that were used to determine viral burden in the CNS by qRT-PCR were used). qRT-PCR was performed to measure the mRNA expression levels of IL-6 (A), CXCL10 (B), IFN-γ (C), IL-1β (D), granzyme B (E), and IL-10 (F). Differences in mRNA expression levels were determined by 2^−ΔΔ^*^CT^* analysis. Expression levels are displayed as Log_2_ fold increases over expression of the cytokine in the brains of naive Ifnar1^−/−^ mice. Statistical significance was determined by Mann-Whitney test (*, *P* = 0.03; **, *P* = 0.002; ***, *P* = 0.0002; ****, *P* < 0.0001). (G) Heat map displaying mean cytokine expression data for each group represented as a percent of the maximum expression level for a given cytokine.

In murine and nonhuman primate models of ZIKV infection, immune cell infiltration in the CNS has been linked to neuronal damage and pathogenesis (44–46). In addition, studies evaluating differences in pathogenicity between different ZIKV lineages have linked expression of multiple cytokines and chemokines in the brain to more severe disease (47). To determine if this could be an underlying contributor to the altered pathogenesis that we observed during heterologous infection, we measured mRNA expression levels of various inflammatory cytokines in the brains of these mice at day 8 post ZIKV challenge (Fig. 5). This cytokine mRNA expression analysis was done using the same RNA extracts that were used to quantify viral burden in the brain (Fig. 4D). Relative to expression levels in the brains of naive Ifnar1^−/−^ mice, we observed an induction in mRNA expression of inflammatory cytokines, such as interleukin-6 (IL-6) (Fig. 5A), CXCL10 (Fig. 5B), and gamma interferon (IFN-γ) (Fig. 5C) in addition to the antiviral molecule IL-1β (48) (Fig. 5D), granzyme B (Fig. 5E), and IL-10 (Fig. 5F). Consistent with our observed pattern of pathogenesis in this heterologous infection system, each cytokine was induced to the greatest extent during primary ZIKV infection (flavi-naive × ZIKV) (Fig. 5G and Table 3), followed by mice with prior DENV3 or YF-17D exposure challenged with ZIKV (DENV3 × ZIKV or YF-17D × ZIKV), and only minimal cytokine induction was observed in mice with prior ZIKV exposure or DENV2 exposure (ZIKV × ZIKV or DENV2 × ZIKV). Overall, this finding is consistent with the literature linking inflammation in the CNS with pathogenesis during primary ZIKV infection and demonstrates that enhanced pathogenesis in murine models of heterologous ZIKV infection is linked with levels of inflammatory cytokines in the CNS.

**TABLE 3:**
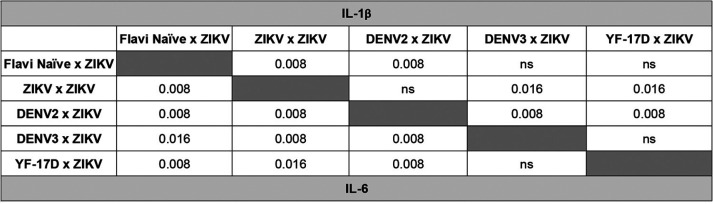
Statistical comparison of IL-1β and IL-6 cytokine production between infected groups of mice as measured by qRT-PCR and ΔΔ*CT* analysis in the brains at day 8 post ZIKV challenge^*a*^

a Cytokine load was compared between each group by Mann-Whitney test. Significant *P* values are indicated for comparison between the groups; ns is used to denote the comparison were the *P* value was above 0.05.

### Defining the relationships of multiple disease metrics during heterologous ZIKV challenge.

**FIG 6:**
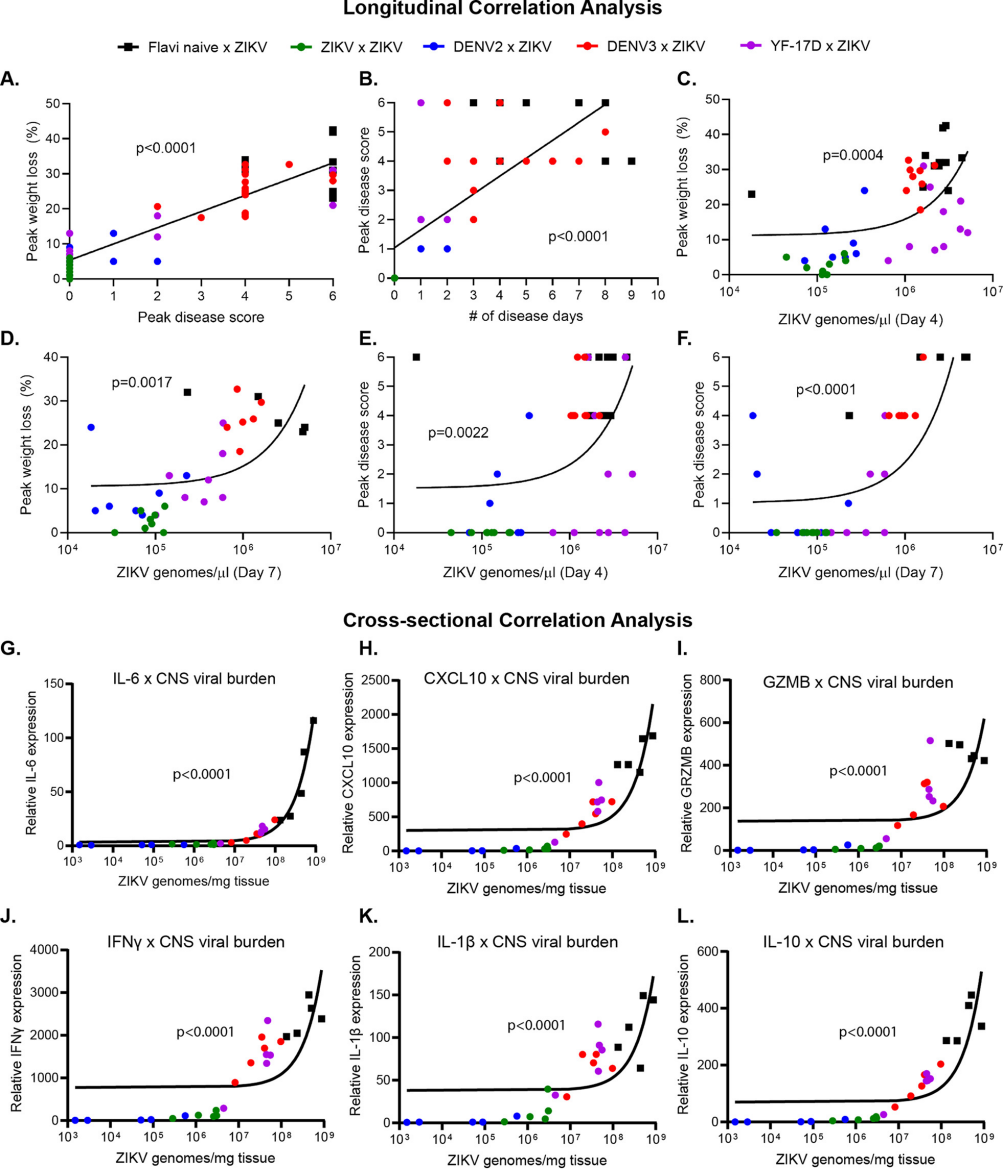
Multiple metrics of ZIKV infection and disease burden are correlated. The data collected from Fig. 2 and 3 were used to generate a data bank and analyzed using linear regression and Pearson correlation analysis to define the relationships of multiple disease metrics during heterologous ZIKV challenge. (A) Linear regression and correlation between the peak weight loss of each animal and peak in disease score. Mice were weighed daily and observed for clinical signs of disease. Each phenotype was assigned a number from 0 to 6 (0 = no disease, 1 = limp tail, 2 = hind limb weakness, 3 = single hind limb paralysis, 4 = bilateral hind limb paralysis, 5= full body weakness/paralysis, and 6 = death). (B) Linear regression and correlation between peak disease score and the number of days a given animal experienced clinical signs of neurological disease. (C) Linear regression and correlation between ZIKV genomes detected in the blood by qRT-PCR on day 4 post ZIKV infection and peak weight loss. (D) Linear regression and correlation between ZIKV genomes detected in the blood by qRT-PCR on day 7 post ZIKV infection and peak weight loss. (E) Linear regression and correlation between ZIKV genomes detected in the blood by qRT-PCR on day 4 post ZIKV infection and peak disease score. (F) Linear regression and correlation between ZIKV genomes detected in the blood by qRT-PCR on day 7 post ZIKV infection and peak disease score. (G–L) The correlative relationship between viral burden in the brain (as measured by qRT-PCR on day 8 postinfection) and inflammatory cytokine expression (as measured by qRT-PCR on day 8 postinfection) in the brain by linear regression and Pearson analysis. IL-6 expression (G), CXCL10 expression (H), granzyme b expression (I), IFN-γ expression (J), IL-1β expression (K), and IL-10 expression (L).

Disease metrics assessed during ZIKV infection in mouse models are diverse. Studies using these models (including our own) have quantified disease using noninvasive techniques, such as weight loss, neurological disease assessment, mortality, viremia, and viral shedding in the urine, as well as more invasive techniques, such as viral burden in multiple target organs, fetal resorption and loss, and neuroinvasion, inflammation, and apoptosis histologically (22–24, 26, 33, 43, 49–52). However, it is unclear how each disease parameter in this complex system is related to the others and whether this is influenced by prior heterologous flavivirus exposure. Therefore, we generated a data bank using longitudinal data points from variables measured in Fig. 2 and 3 from each individual mouse. We used these data to determine correlative relationships between various metrics of disease, including peak percent weight loss, day of peak weight loss, peak disease score, day of peak disease, number of days of disease, and viremia on days 4, 7, 14, and 30 post ZIKV infection by linear regression and Pearson correlation (Fig. 6A to F and Table 4). The peak weight loss percentage was determined by normalizing the starting weights of each animal at day 0 to 100% and assessing weight daily and noting the peak percentage of weight lost through the course of infection. The peak disease score was determined by tracking neurological indicators as previously described daily following ZIKV infection (22, 23). Each indicator of disease was assigned a number from 0 to 6, indicative of severity (0 = no disease, 1 = limp tail, 2 = hind limb weakness, 3 = single hind limb paralysis, 4 = bilateral hind limb paralysis, 5 = full body weakness/paralysis, and 6 = death). The peak day of disease corresponded to the day of peak neurological disease based on this scoring system. The number of disease days was determined by counting each day for each mouse that the disease score was above 0 and ending at the time of death if applicable.

**TABLE 4:**
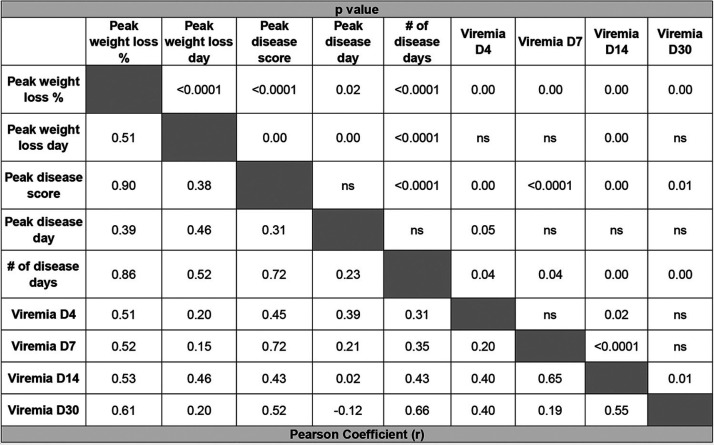
Correlation matrix defining the relationship between multiple disease metrics in a longitudinal heterologous infection study^*a*^

a The data collected from Fig. 2 and 3 were used to generate a data bank and analyzed using linear regression and Pearson correlation analysis to define the relationships of the coefficient (*r*) (lower left) demonstrating the strength and directionality of the correlation between each variable and the *P* value (upper right) demonstrating the statistical significance of each correlation.

Of the 36 bivariate permutations, we identified 25 statistically significant correlative interactions. The strength and directionality of each correlation are indicated by the Pearson coefficient (*r*), and the statistical significance of each correlation is indicated by the *P* value (Table 4). From these data, we identified several interactions of particular interest (Fig. 6A to F). The strongest correlation resulted when comparing the peak neurological disease score from each mouse to the peak percent weight lost (*r* = 0.8973; *P* < 0.0001) (Fig. 6A). As one might expect, as the peak neurological disease score identified in each animal increased, so did the amount of weight lost. Importantly, each infection group (flavi-naive × ZIKV, DENV2 × ZIKV, DENV3 × ZIKV, YF-17D × ZIKV, and ZIKV × ZIKV) generally clustered together and along the pattern determined by linear regression. This would suggest similarities in the biological characteristics of these groups. We were also interested in the relationship between the number of days each infected animal displayed a neurological disease phenotype and the severity of the disease phenotype (Fig. 6B). We found that the peak disease score and the number of measured disease days strongly positively correlated with one another (*r* = 0.7234; *P* < 0.0001); that is, in general, the more severe the disease phenotype, the longer it would take to resolve.

We were particularly interested in the relationship between viremia and less invasive metrics of disease burden, such as weight loss and disease score. When comparing viral burden on day 4 or day 7 to peak percentage of weight loss (Fig. 6C and D, respectively), we found a statistically significant positive correlation (*r* = 0.5078 and 0.5184 and *P* = 0.0004 and 0.0017, respectively). This was also true when comparing viral burden on day 4 or day 7 to peak disease score (Fig. 6E and F) (*r* = 0.45 and 0.7213 and *P* = 0.022 and <0.0001, respectively). That is, with increased viremia on these days came increased weight loss and neurological disease throughout the course of infection. The correlative analyses associating disease severity and viral load at day 4 are of particular interest due to the timing. As demonstrated in Fig. 2, neurological indicators of disease are not overtly detectable until day 5 postinfection and typically do not peak until days 7 to 9 (Fig. 2D), and the most significant drop in weight also occurs from days 7 to 9 (Fig. 2C). However, using the disease metric of early viremia on day 4, it is clear that information could be used in linear regression analysis to predict the severity and outcome of infection days earlier than the occurrence of overt disease (Fig. 6C and 6E).

Finally, in order to understand the relationship between viral burden and inflammation as a metric of disease in the context of heterologous infection, we compared the viral burden in the brain at day 8 post ZIKV challenge (Fig. 4D) and cytokine expression levels in the brain at day 8 post ZIKV challenge in the same animals (Fig. 5). We used these data to determine correlative relationships between CNS viral load and cytokine expression during ZIKV challenge by linear regression and Pearson correlation analysis (Fig. 6G to L and Table 5). For each cytokine that was analyzed (IL-6, CXCL10, granzyme B, IFN-γ, IL-1β, and IL-10), we observed a pronounced and statistically significant positive correlation between its expression level and viral burden in the CNS (*r* = 0.9808, 0.9815, 0.9231, 0.9585, 0.9123, and 0.9862, respectively) (Fig. 6G to L). The strength of the correlation of viral burden and cytokine expression in the CNS during ZIKV infection is consistent with previous studies that have linked ZIKV-induced disease to enhanced expression of proinflammatory cytokines in the CNS (Table 5) (47). Overall, these data demonstrate the bivariate interactions between various metrics that are commonly used to assess ZIKV disease burden in mouse models of infection and importantly show that, in the context of heterologous infection, these correlations are still appropriate and comparable.

**TABLE 5:**
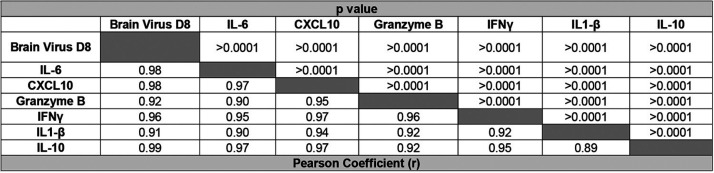
The correlative relationship between viral burden in the brain and inflammatory cytokine expression^*a*^

a Both cytokine expression and viral burden were measured by qRT-PCR on day 8 postinfection in the brains of the infected animals. Linear regression and Pearson correlation analysis were used to define the relationships of the coefficient (*r*) (lower left) demonstrating the strength and directionality of the correlation between each variable and the *P* value (upper right) demonstrating the statistical significance of each correlation.

## DISCUSSION

Increased globalization, deforestation, climate change, and the lack of effective vaccines has resulted in most of the world’s population being at risk for infection with multiple flaviviruses (53). There is no vaccine available for ZIKV, and current vaccines for flaviviruses including the yellow fever vaccine, while highly effective, have not prevented outbreaks from these highly prevalent arboviruses. While the current number of ZIKV cases in the Americas has dropped significantly compared to that in 2016, based on the infection cycles of similar flaviviruses, it is believed that ZIKV will follow a similar cyclical pattern of emergence and reemergence (54, 55). Therefore, it is highly likely that ZIKV is in an interepidemic period and will reemerge and continue to spread throughout the Americas as has been seen with both DENV1 to 4 and YFV.

The influence of prior flavivirus exposure on ZIKV protection and pathogenesis remains an important question. Epidemiological studies do provide some insight into these competing concerns. A study in Brazil comparing YFV vaccination coverage with incidence rates of ZIKV-associated microcephaly found that Northeast Brazil, which had the highest incidence of ZIKV-associated microcephaly, also had relatively low YFV vaccination rates, suggesting that a lack of YFV vaccination left that population without a cross-protective response and, therefore, they were more susceptible to ZIKV morbidity (31). Moreover, the Harris group has examined the relationship between prior DENV exposure and the incidence of asymptomatic ZIKV infection in a pediatric cohort in Nicaragua, finding that children with prior DENV infection had lower rates of symptomatic ZIKV infection, again suggesting ZIKV cross-protection was mediated by previous DENV exposure (29). However, drawing clear causal links between previous exposure and infection outcomes are a challenge in human populations for several reasons in this case. These cocirculating flaviviruses share antigenic similarities, which can confound many serologically based diagnostic tests, which makes confirming records of the natural history of infection particularly challenging. Additionally, the length of time between exposures of heterologous serotypes of DENV plays a major role in whether increased incidence of enhanced pathogenesis or cross-protection occurs (18, 56, 57).

In this study, we challenged the hypothesis that prior heterologous flavivirus exposure to DENV serotypes 2 or 3 or YF-17D would confer equal cross-protection from ZIKV in a mouse model. We ultimately showed that a sublethal heterologous flavivirus exposure confers varying degrees of protection from ZIKV mortality, weight loss, and neurological disease. Prior exposure to ZIKV or DENV2 was the most protective from ZIKV challenge, with no mice succumbing to infection and few, if any, displaying any signs of neurological disease and weight loss (Fig. 2). Exposure to YF-17D or DENV3 lessened mortality, disease severity, and viral burden, though some animals still succumbed to infection. Importantly, prior exposure to either ZIKV, DENV2, DENV3, or YF-17D significantly reduced viral burden in the spleen, liver, kidney, brain, and spinal cord of mice infected with ZIKV (Fig. 4). These data demonstrate a cross-protective effect of prior flavivirus exposure on ZIKV replication and disease burden.

When analyzing ZIKV neutralization potential of polyclonal serum samples from mice with prior flavivirus exposure during ZIKV infection, we found that prior exposure to ZIKV resulted in high levels of circulating neutralizing antibodies that persisted during ZIKV challenge (Fig. 3E and F). However, mice with prior heterologous flavivirus exposure did not have detectable neutralizing antibodies to ZIKV prior to ZIKV challenge. This demonstrates that the mechanism of this observed cross-protection is not likely mediated by ZIKV cross-neutralizing antibodies. However, these studies are limited in that they did not address the potential for other mechanisms of cross-protection, including nonneutralizing antibody functions (58) or the presence of cross-reactive T cells (25, 59).

Murine studies have linked inflammation in the brain to increased ZIKV pathogenesis (47). We found that prior heterologous flavivirus exposure conferred varying degrees of cross-protection from ZIKV pathogenesis that was dependent upon the identity of the primary infecting virus. Based on the established link between inflammation and ZIKV pathogenesis in a primary ZIKV infection, we hypothesized that the different levels of pathogenesis that we observed in this system would associate with the induction of cytokine expression in the brains of these mice. We found that in groups that displayed elevated pathogenesis (DENV3 × ZIKV or YF-17D × ZIKV), there were higher levels of inflammatory transcripts in the brain relative to those of groups that displayed minimal pathogenesis (ZIKV × ZIKV or DENV2 × ZIKV) (Fig. 5). Importantly, we found that the level of inflammatory cytokine induction strongly correlated with viral burden in the CNS of these animals (Fig. 6G to L), which demonstrates that, even in the added context of heterologous infection, the established correlation between ZIKV-induced disease and inflammatory cytokine expression is appropriate and comparable to a primary ZIKV infection. These findings provide insight into the cause of the variable degrees of ZIKV cross-protection observed.

Murine models of ZIKV infection and heterologous flavivirus challenge have used diverse metrics for quantifying disease burden (22–24, 26, 33, 43, 49–52). Until now, the relationship of many of these variables has not been evaluated. In this longitudinal heterologous challenge experiment, we performed linear regression and correlation analysis to determine the relationship between multiple variables in individual mice, including peak weight loss, day of peak weight loss, peak disease score, day of peak disease score, number of disease days, and viremia on days 4, 7, 14, and 30. Within this longitudinal analysis, we identified 25 statistically significant correlative interactions (Table 4). Importantly, we found that viral burden on day 4 strongly correlated with the peak weight loss and peak disease score that an animal would eventually experience (typically on days 7 to 9 postinfection) (Fig. 6C and E). This allows for the possibility of using early viremia data as a predictor of severe disease outcomes using linear regression analysis that can also be applied in the context of heterologous infection scenarios.

Ultimately, these data provide additional evidence of the cross-protective effect of prior heterologous flavivirus exposure on ZIKV disease. These findings are important given that the majority of the world is at risk of flavivirus exposure and multiple flaviviruses are endemic in many regions. Addressing this is not only important for being able to predict the outcome of ZIKV exposure in areas of flavivirus endemicity but will support efforts to generate a pan-flavivirus vaccine. While this study provides significant insight into cross-protection from ZIKV, additional studies are desperately needed to understand the mechanism behind this. Studies such as these will be essential to control these significant public health threats.

## MATERIALS AND METHODS

### Ethics statement.

All animal studies were done in accordance with the Guide for Care and Use of Laboratory Animals of the National Institutes of Health and approved by the Saint Louis University Animal Care and Use Committee (IACUC protocol number 2667).

### Viruses and cells.

P_0_ stocks of ZIKV strain PRVABC59 (GenBank accession number KU501215.1) and YFV strain 17D (GenBank accession number X03700) were acquired from Biodefense and Emerging Infection (BEI) Resources. Each virus was passaged in African green monkey kidney epithelial cells (Vero-WHO) that were purchased from the American Type Culture Collection (ATCC CCL-81). The supernatants of these cultures were clarified of cellular debris by centrifugation at 3,500 rpm prior to being aliquoted and frozen at −80°C. DENV2 strain D2S20 (GenBank accession number HQ891024) was a kind gift from Michael Diamond (60). DENV3 strain C0360/94 (GenBank accession number AY923865) was obtained from ATCC. Both DENV2 and DENV3 were grown in C6/36 Aedes albopictus cells (ATCC CRL-1660). At the time of harvest, the medium supernatant was clarified of cellular debris by centrifugation at 3,500 rpm. Each virus was then concentrated by ultracentrifugation at 30,000 rpm over a 25% glycerol cushion before being aliquoted and frozen at −80°C (19). The infectious titer of each viral stock was quantified by focus forming assay (FFA) as previously described (49). Briefly, a 90% confluent monolayer or Vero-WHO cells was plated in a 96-well flat-bottom plate. Serial dilutions of each viral stock were added to each well for 1 h, prior to the addition of a methyl cellulose layer, to restrict lateral spread of the virus. After 48 h (ZIKV and YF-17D) or 72 h (DENV2 and DENV3), the cells were fixed and permeabilized. The cells were incubated with a flavivirus cross-reactive monoclonal primary antibody (4G2) for 1 h at room temperature, washed, incubated with a horseradish peroxidase conjugated anti-mouse secondary antibody for 1 h at room temperature, and washed. Foci of infected cells were visualized and quantified following the addition of True-Blue peroxidase substrate.

### Mice and infections.

IFN-α/β receptor 1 knockout (Ifnar1^−/−^) mice were purchased from Jackson Laboratories. They were bred and maintained at Saint Louis University in a specific pathogen-free mouse facility. To achieve a primary infection, at 4 to 5 weeks of age, equal ratios of male and female mice were administered a sublethal intravenous (i.v.) challenge of either DENV2 (10^5^ FFU) or DENV3 (10^5^ FFU). To generate mice with prior ZIKV or YF-17D exposure, 8-week-old mice at equal ratios of male and female animals, were administered a sublethal subcutaneous (s.c.) challenge of either ZIKV (10^5^ FFU) or YF-17D (10^5^ FFU). The viral doses, routes of administration, and ages of mice were deliberately chosen based on optimized dosing experiments in our lab known to induce detectable viral replication and immune responses but not cause mortality (19, 21–23). As a flavivirus-naive group, 8-week-old littermate controls were administered PBS. At least 30 days following primary challenge, mice were administered an i.v. ZIKV challenge (10^5^ FFU), previously demonstrated by our lab to result in severe neurological sequela and weight loss in 100% of adult Ifnar1^−/−^ mice with no prior flavivirus exposure, with 80 to 100% ultimately succumbing to infection (22, 23). Following ZIKV challenge, mice were monitored daily for 14 days for weight loss, indicators of neurological disease, and mortality. Whole blood was collected longitudinally from each mouse at day 0, 4, 7, 14, and 30 to monitor peripheral viral burden or at day 0 and 14 to assess neutralizing antibody responses from serum.

### Measurement of viral burden.

For longitudinal studies, whole blood was collected by cheek bleed into EDTA coated tubes. Fifty microliters of blood was transferred to RNAsol BD reagent, and RNA was extracted according to the manufacturer’s instructions. For studies evaluating global viral burden, on day 4 and 8 post ZIKV infection, mice were administered a lethal cocktail of ketamine/xylazine before intracardiac perfusion with 20 ml of PBS. The spleen, liver, kidney, brain, and spinal cord were collected from each mouse and snap-frozen in a dry ice bath. Organs were weighed and homogenized in DMEM using a Bead Mill 24 from Fisher Scientific. RNA was extracted from 100 μl of homogenate using Tri Reagent RT according to the manufacturer’s instructions. ZIKV RNA was quantified by qRT-PCR using a PrimeTime primer-probe set (forward, CCGCTGCCCAACACAAG; reverse, CCACTAACGTTCTTTTGCAGACAT; probe, AGCCTACCTTGACAAGCAGTCAGACACTCAA) and an in-house ZIKV RNA copy control. To measure infectious virus, spleen, and brain homogenate on day 4 or 8 post ZIKV, challenge was serially diluted and utilized in a standard focus forming assay (FFA) as previously described (49).

### ZIKV FRNT.

The ability of murine polyclonal serum samples to neutralize ZIKV was measured by a focus reduction neutralization test (FRNT). Briefly, murine serum was serially diluted and incubated with a consistent quantity of infectious ZIKV at 37°C for 1 h to allow for complex formation. Following incubation, the virus-serum complex was added to each well of a 96-well plate containing a confluent monolayer of Vero-WHO cells. Following a 1-h incubation to allow for attachment, a methyl cellulose overlay was added to restrict lateral viral spread. Following 2 days of infection, the cell monolayer was fixed and infectious virus was quantified by a standard FFA, as previously described (49). Neutralization potential is displayed as 1/NT_50_ with higher values indicating higher neutralization potential.

### Measurement of cytokine expression.

mRNA expression of IL-6, CXCL10, granzyme B, IFN-γ, IL-1β, and IL-10 was determined in RNA extracts from brains 8 days postinfection by qRT-PCR using TaqMan primer probe sets from Integrated DNA Technologies (IDT) (assay identifiers—Mm.PT.58.41769240 [IFNG], Mm.PT.58.10005566 [IL-6], Mm.PT.58.42155916 [GZMB], Mm.PT.58.41616450 [IL-1β], Mm.PT.58.43575827 [CXCL10], and Mm.PT.58.13531087 [IL-10]). Relative expression for each cytokine was determined by 2^−ΔΔ^*^CT^* analysis with fold induction being relative to cytokine levels in brain RNA extracts of naive Ifnar1^−/−^ mice.

### Statistical analysis.

For DENV, ZIKV, YFV, and YF-17D incidence maps, incidence data and vaccine coverage data were collected for South and Central American countries from the WHO/PAHO for the years 2015 to 2019 and displayed as the annual average number of cases per 100,000 individuals using the spatial data program GeoDa (61). Amino acid identity for each flavivirus of interest was determined by performing a global alignment allowing for free ends using a Blosum62 cost matrix in the software Geneious. Statistical analyses for *in vivo* studies were performed using GraphPad Prism. Statistical differences in survival were determined using a Mantel-Cox test. Differences in weight loss and viral burden over time were determined using a two-way analysis of variance (ANOVA) with *post hoc* analysis. Statistical significance in viral burden and cytokine expression in various organs was determined by Mann-Whitney test. Correlative analysis was performed using linear regression analysis and a two-tailed Pearson analysis. Statistical significance has been indicated within the figures with asterisks (*, *P* = 0.03; **, *P* = 0.002; ***, *P* = 0.0002; ****, *P* < 0.0001).

### Data availability.

Data are available online (62–65).
